# A tiny, long-legged raptor from the early Oligocene of Poland may be the earliest bird-eating diurnal bird of prey

**DOI:** 10.1007/s00114-020-01703-z

**Published:** 2020-10-08

**Authors:** Gerald Mayr, Jørn H. Hurum

**Affiliations:** 1 Ornithological Section, Senckenberg Research Institute and Natural History Museum Frankfurt, Senckenberganlage 25, D-60325 Frankfurt am Main, Germany; 2 Natural History Museum, University of Oslo, P.O. 1172 Blindern, 0318 Oslo, Norway

**Keywords:** Accipitridae, Aves, Evolution, Fossil birds, Rupelian

## Abstract

We report a small hawk-like diurnal bird from the early Oligocene (30–31 million years ago) of Poland. *Aviraptor longicrus*, n. gen. et sp. is of a size comparable with the smallest extant Accipitridae. The new species is characterized by very long legs, which, together with the small size, suggest an avivorous (bird-eating) feeding behavior. Overall, the new species resembles extant sparrowhawks (*Accipiter* spp.) in the length proportions of the major limb bones, even though some features indicate that it convergently acquired an *Accipiter*-like morphology. Most specialized avivores amongst extant accipitrids belong to the taxon *Accipiter* and predominantly predate small forest passerines; the smallest *Accipiter* species also hunts hummingbirds. Occurrence of a possibly avivorous raptor in the early Oligocene of Europe is particularly notable because *A. longicrus* coexisted with the earliest Northern Hemispheric passerines and modern-type hummingbirds. We therefore hypothesize that the diversification of these birds towards the early Oligocene may have triggered the evolution of small-sized avivorous raptors, and the new fossil may exemplify one of the earliest examples of avian predator/prey coevolution.

**Electronic supplementary material:**

The online version of this article (10.1007/s00114-020-01703-z) contains supplementary material, which is available to authorized users.

## Introduction

Accipitrid diurnal birds of prey include 237 extant species with diverse ecological preferences and feeding habits (Thiollay 1994). Well-resolved molecular phylogenies (Lerner and Mindell 2005; Griffiths et al. 2007; Mindell et al. 2018) show that various small clades with geographically restricted distributions diverged early in the phylogeny of the Accipitridae, whereas the species-rich taxa Aquilinae (eagles and allies), Buteoninae (buzzards and allies), and Accipitrinae (sparrowhawks, harriers, and allies) form a deeply nested clade.

Diurnal birds of prey have a scant early Cenozoic (Paleogene) fossil record and most aspects of their evolutionary history therefore remain poorly known (Mayr 2009, 2017). The oldest fossil is from the early Eocene (50.5–52 million years ago [Ma]) of Belgium (Mayr and Smith 2019), but this specimen—a tarsometatarsus fragment—cannot be assigned to a particular accipitrid clade and does not provide insights into the ecology and feeding habits of the unnamed species it belonged to. The same is true for most other Paleogene remains of diurnal birds of prey described so far (Harrison and Walker 1979; Mourer-Chauviré 1991; Mayr and Smith 2002). The few species known from more diagnostic elements (mainly tarsometatarsi) represent medium-sized to larger “buzzard”- or “eagle”-type species, which presumably foraged on small mammals or other terrestrial vertebrates; most of these fossils are from the late Eocene and early Oligocene of North America (Mayr and Perner 2020).

Here we describe a skeleton of a small raptor from the early Oligocene of Poland, which resembles sparrowhawks (*Accipiter* spp.) in the length proportions of the limb bones and represents a distinctive accipitrid morphotype that has not yet been reported from the Paleogene period. The fossil is from early Oligocene (Rupelian) strata of the locality Jamna Dolna 2 in southeast Poland, a site that has already yielded a few other bird remains (Bochenski et al. 2011; Mayr and Bochenski 2016; Mayr 2019; Mayr et al. in press). The new fossil is characterized by very long legs, which indicate an avivorous (bird-eating) feeding behavior. As such, it not only sheds light on the evolutionary history and past diversity of the Accipitridae but may also contribute to an understanding of early Oligocene bird communities.

## Material and methods

The fossil is deposited in the paleontological collections of the Natural History Museum, Oslo, Norway (PMO). Comparisons with extant Accipitridae are based on skeletons in the collection of Senckenberg Research Institute Frankfurt (SMF). To aid anatomical descriptions, UV-induced fluorescence photographs of the fossil were taken.

## Results

Aves Linnaeus, 1758

Accipitriformes Vieillot, 1816

Accipitridae Vigors, 1824

*Aviraptor longicrus*, n. gen. et sp.

### Holotype

**Fig. 1:**
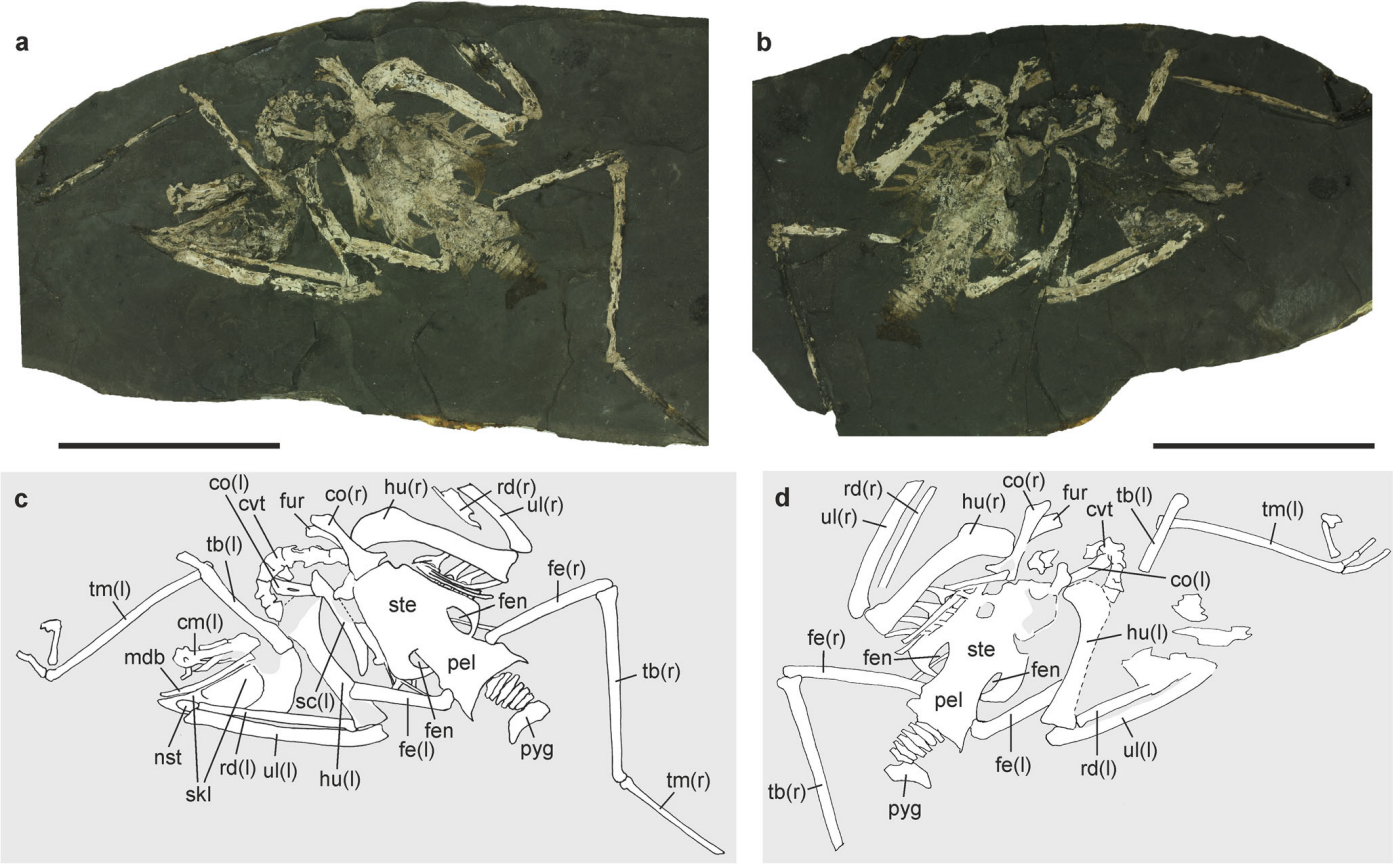
**a***–***d** Holotype of *Aviraptor longicrus*, n. gen. et sp. from the early Oligocene of Poland with interpretive drawings (**a**, **c**: specimen PMO 234.584a; **b**, **d**: specimen PMO 234.584b). *cvt* cervical vertebrae, *fen* sternal fenestrae, *fur* furcula, *cm* carpometacarpus, *co* coracoid, *fe* femur, *hu* humerus, *mdb* mandible, *nst* nostril, *pel* pelvis, *pyg* pygostyle, *rd* radius, *sc* scapula, *skl* skull, *ste* sternum, *tb* tibiotarsus, *tm* tarsometatarsus, *ul* ulna. For limb bones, left (l) and right (r) sides are indicated. Scale bars equal 50 mm. [Color online]

PMO 234.584a + b (skeleton on two slabs, lacking the distal portion of the right wing and most of the toes of both feet; Fig. 1).

### Diagnosis

Very small accipitrid that is characterized by a greatly elongated tarsometatarsus and short and stocky humerus, with humerus: tarsometatarsus ratio being 0.86 (≥ 0.9 in extant Accipitridae); beak only slightly hooked; sternum about as wide as long and with pair of large fenestrae in caudal margin; ulna and tibiotarsus subequal in length; proximal phalanx of second toe not strongly abbreviated.

### Differential diagnosis

Distinguished from all previously described Paleogene Accipitridae by small size and long and slender tarsometatarsus. Differs from the late Eocene Horusornithidae in larger size, more elongate and gracile tarsometatarsus, and humerus with proportionally wider proximal end and without prominent dorsal supracondylar tubercle on distal end. Distinguished from all extant Accipitridae except some species of *Accipiter* in small size and long tarsometatarsus; differentiated from *Accipiter* spp. by absence of concave notch in proximal portion of deltopectoral crest and proportionally longer proximal phalanx of second toe.

### Type locality and horizon

Jamna Dolna 2, southeast of Bircza, Subcarpathian Province, southeast Poland; early Oligocene, Rupelian (nannoplankton zone NP 23), black shales of the Menilite beds of the Carpathian Flysch zone exposed on the eastern side of the Jamninka river, presumably local ichthyofaunal zone IPM 3 (30–31 Ma) (Bieńkowska-Wasiluk 2010).

### Measurements (left/right, maximum length in mm)

Skull, 36.5; upper beak, ~ 11.3; humerus, −/37.5; ulna, 47.2/−; femur, −/31.8; tibiotarsus, −/45.3; tarsometatarsus, 43.8/−.

### Etymology

The genus name is coined from avis (Lat.): bird and raptor (Lat.): thief, in reference to the presumed avivorous (bird-eating) habits of the new species. The species epithet is derived from the Latin words longus (long) and crus (leg).

### Description and comparison

**Fig. 2:**
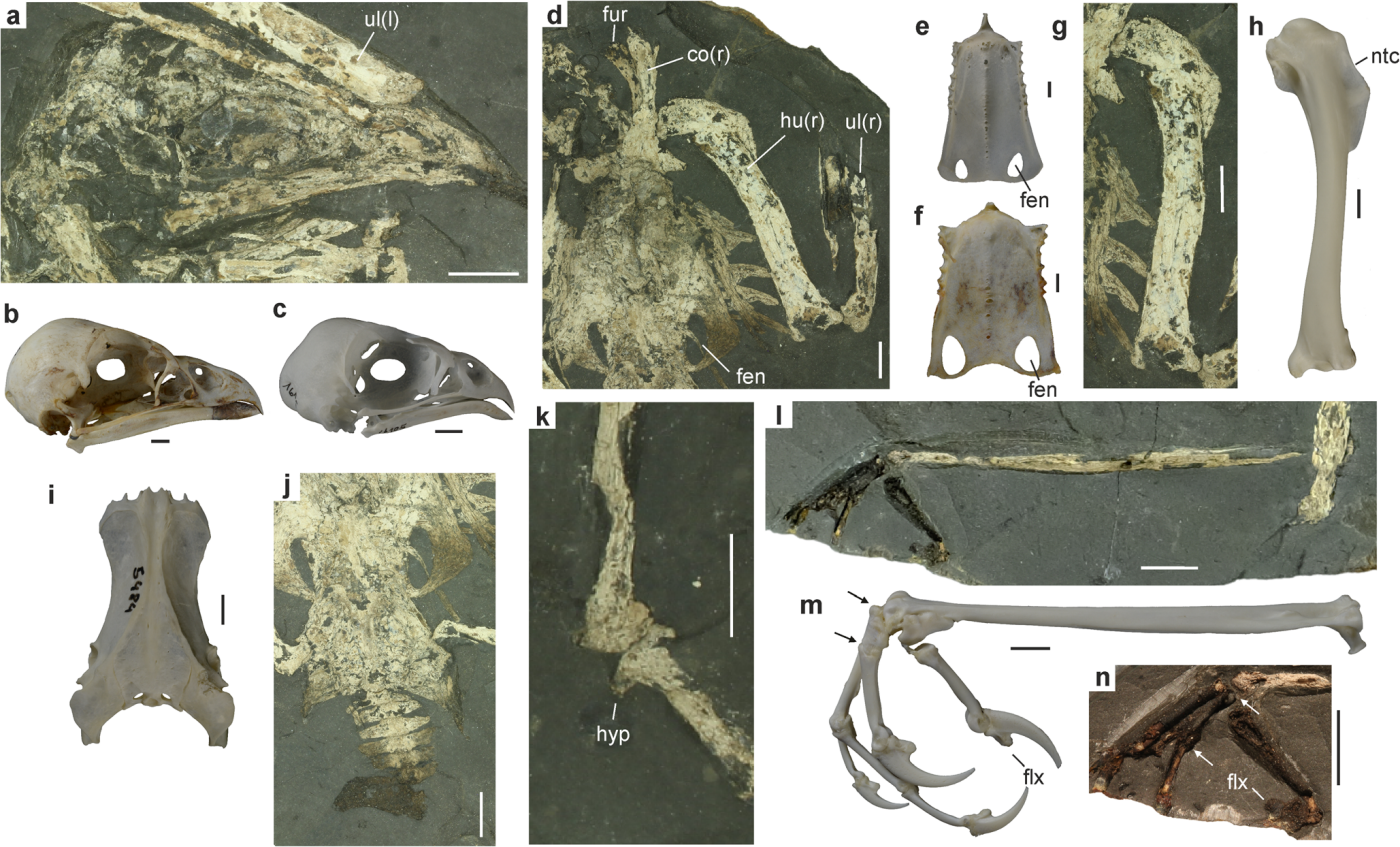
**a** Skull of *Aviraptor longicrus*, n. gen. et sp. from the early Oligocene of Poland (PMO 234.584a). **b**, **c** skulls of **b** *Polyboroides typus* and **c** *Accipiter nisus*. **d** pectoral region with sternum and humerus (PMO 234.584a). **e**, **f** sternum of **e** *A. nisus* and **f** *Butastur rufipennis*. **g**, **h** right humerus of the fossil (PMO 234.584a) and *A. nisus*. **i** pelvis of *A nisus*. **j** pelvis and caudal vertebrae of the fossil (PMO 234.584a). **k** right intertarsal joint of the fossil (PMO 234.584a). **l**, **m** left foot of the fossil (PMO 234.584b) and right foot of *A. nisus*. **n** detail of left foot of the fossil (PMO 234.584b). Arrows in **m** and **n** delimit the proximal phalanx of the second toe. *fen* sternal fenestrae, *flx* flexor tubercle, *fur* furcula, *co* coracoid, *hu* humerus, *hyp* hypotarsus, *ntc* concave notch in deltopectoral crest, *ul* ulna. For limb bones, left (l) and right (r) sides are indicated. *Scale bars* equal 5 mm. [Color online]

In its relative size and overall shape, the comparatively small skull resembles that of extant hawk-like Accipitridae. The upper beak is short and measures less than one fourth of the entire skull length (Fig. 2a). Unlike in most extant Accipitridae (Fig. 2c), its tip lacks a sharply deflected hook, but an equally weakly deflected tip of the beak occurs in the accipitrid taxon *Polyboroides* (Fig. 2b). The ovate nostril is large; as in extant Accipitridae and Falconidae, the internarial septum is extensively ossified. Osteological details of the neurocranium cannot be discerned owing to the substantial crushing of the bones. The depth of the mandible is similar to that of most hawks (e.g., *Accipiter* spp.); unlike in the Falconidae, there is no mandibular fenestra.

Nine short cervical vertebrae are visible in the fossil, but these do not allow a meaningful description. There are six free caudal vertebrae. The pygostyle resembles that of *Accipiter* spp. in its shape; the lamina has a concave caudal margin.

The sternum is about as wide as it is long and exhibits a pair of large fenestrae in its caudal portion. In its proportions, it resembles that of *Butastur rufipennis* (Fig. 2f), whereas the sternum of the Accipitrinae (Fig. 2e) is more elongated. Five sternal ribs can be discerned. The uncinate processes of the vertebral ribs are co-ossified with the ribs.

The right coracoid is well exposed and has a bulky omal extremity and a narrow lateral process (Fig. 2d). Whether a foramen for the supracoracoideus nerve is present cannot be discerned (this foramen is absent in the species of *Accipiter* and *Circus*, but present in most other Accipitridae). The scapula has a well-developed acromion with a broad tip and its blade widens towards the caudal end of the bone. The omal extremity of the furcula is short and has a blunt tip.

The humerus is robust and stocky and even though taphonomic compression of the bone may account for some widening of the shaft, its relative width appears to be greater than in any extant Accipitridae and Falconidae. The deltopectoral crest is restricted to the proximal third of the bone and has a rounded profile, whereas its proximal portion forms a shallow concave notch in *Accipiter* and most other extant Accipitridae (Fig. 2g, h). Furthermore unlike in *Accipiter*, the tuberculum dorsale is not proximodorsally projected. On the distal end of the bone, a distomedially prominent flexor processus is absent.

The ulna exceeds the humerus in length. The proximal end of the radius exhibits a wide ulnar articulation facet, which—as in extant Accipitridae—projects distinctly beyond the margins of the shaft. The hand section of the left wing is detached from the ulna and radius and situated ventral of the mandible. Of the carpometacarpus, only the proximal half is exposed and the profile of the caudal rim of the carpal trochlea is only weakly convex.

The pelvis (Fig. 2j) has similar proportions to that of extant Accipitridae (Fig. 2i), with the preacetabular section being about twice as long as the postacetabular one (in most non-raptorial birds both sections are of equal length). The pelvis widens cranially and the preacetabular part has concave lateral margins.

The femur has a low trochanteric crest. The tibiotarsus reaches almost the length of the ulna, whereas it is distinctly shorter than the ulna in most extant diurnal birds of prey (Supplementary Table 1). The tarsometatarsus is particularly long and slender. Unlike in most extant Accipitridae, the greatly elongated tarsometatarsus is distinctly longer than the humerus. In lateral view, its shaft is slightly bowed. The hypotarsus (Fig. 2k) is proximodistally short and offset from the shaft; unlike in the Falconidae, it does not merge into a crista plantaris.

Of the feet, only the hallux and the proximal portions of the three anterior toes are preserved. The ungual phalanx of the long hallux exhibits a well-developed flexor tubercle, which is also found in other diurnal birds of prey (the distal section of the ungual phalanx is not preserved). The proximal phalanx of the second toe is proportionally longer than in all extant Accipitridae (Fig. 2m, n) except for pernine kites (*Pernis*, *Aviceda*).

## Discussion

*Aviraptor longicrus*, n. gen. et sp. closely resembles diurnal birds of prey in its skeletal morphology, with shared derived features including the proportions of the short beak, the presence of a pair of large fenestrae in the caudal margin of the sternum, the narrow processus lateralis and bulky omal extremity of the coracoid, the long preacetabular section of the pelvis, and the long hallux whose claw bears a large tuberculum flexorium. The new species furthermore agrees with the Accipitridae and differs from the superficially similar Falconidae—which originated in the New World and did not occur in Europe before the mid-Cenozoic (Mayr 2017)—in the proximodistally short hypotarsus of the tarsometatarsus (in the Falconidae, the medial hypotarsal crest is continuous with a crest along the plantar surface of the tarsometatarsus), the large and ovate nostril (smaller and circular in the Falconidae), and the absence of a mandibular fenestra.

*Aviraptor longicrus* was about the size of *Falco sparverius* (Falconidae; Supplementary Table 1) and the smallest extant Accipitrinae (*Accipiter superciliosus*, *A. minullus*). Skeletons of the latter two species were not available for comparisons, but the minimum weights of *Accipiter superciliosus* and *A. minullus* are about 90% of those of *A. striatus* (75 g versus 82 g; Thiollay 1994). Because the lengths of the major limb bones of *Aviraptor longicrus* measure about 90% of those of male individuals of *Accipiter striatus* (Supplementary Table 1), we assume that *A. longicrus* was of similar size to *A. superciliosus* and *A. minullus*.

**Fig. 3:**
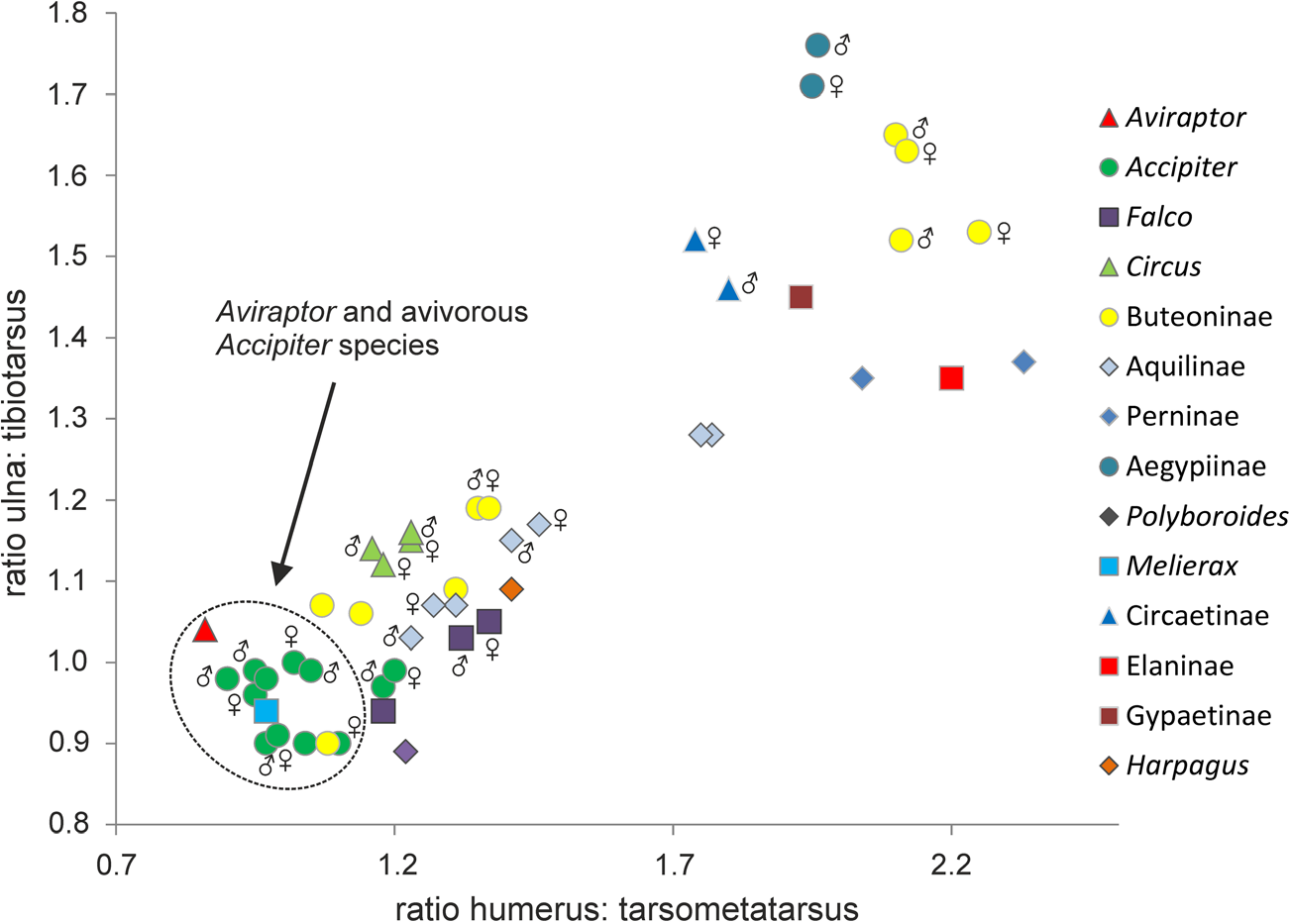
Diagram illustrating ratios of major limb bones of *Aviraptor longicrus*, n. gen. et sp. and extant Accipitridae and Falconidae. *A. longicrus* clusters with avivorous species of *Accipiter* as well as with *Melierax canorus* and *Buteo magnirostris*, which are generalists. Gender symbols denote different sexes of the same species (see Supplementary Table 1 for individual data). [Color online]

The fossil is characterized by very long legs and its limb proportions correspond best with those of some species of *Accipiter* (Fig. 3; Supplementary Table 1). Even though not many osteological details can be discerned in the holotype, *A. longicrus* is distinguished from the species of *Accipiter* and most other extant Accipitridae by a long proximal phalanx of the second toe, which in extant accipitrids is only found in the short-legged pernine kites (*Pernis*, *Aviceda*). In all other accipitrid species, this phalanx is abbreviated, suggesting that *Aviraptor longicrus* probably convergently evolved an *Accipiter*-like morphotype. This is also indicated by differences in the shape of the deltopectoral crest, which has a more rounded profile in the fossil species (Fig. 2g, h).

The assumption that the *Accipiter*-like morphotype is apomorphic for *Aviraptor longicrus* also conforms with calibrated molecular data, which suggest an origin of the Accipitrinae, the accipitrid clade including *Accipiter*, *Circus*, and a few other taxa, in the late early Miocene, about 16–18 Ma (Nagy and Tökölyi 2014; Mindell et al. 2018). The new fossil species is nearly twice as old and predates this divergence estimate by 13–15 million years. The oldest fossils assigned to *Accipiter* are from the early Miocene of Kenya (Walker and Dyke 2006), but the specimens are too fragmentary for unambiguous identification.

Diurnal birds of prey exhibit diverse feeding habits and forage on all kinds of invertebrates and vertebrates, even though mammals constitute the main prey of most species. Often, these disparate dietary preferences are reflected by differences in tarsometatarsus and beak proportions. Very long tarsometatarsi occur in a few highly specialized taxa that prey upon eggs or nestlings of cavity-nesting birds (*Polyboroides*, *Geranospiza*) or in birds of prey with a highly terrestrial way of living, such as the secretarybird (*Sagittarius serpentarius*, Sagittariidae). None of these latter species is, however, as small as *Aviraptor longicrus*. In extant hawk-like diurnal birds of prey, a very small size and long legs are only found in avivorous species predominantly preying on birds, which is true for many representatives of the taxon *Accipiter* (Fig. 3; Thiollay 1994). For *A. longicrus*, the ratio of the length of the upper beak to the total skull length, which is ~ 0.31, also falls within the range of avivorous diurnal birds of prey (0.29 ± 0.03; Hertel 1995). Acknowledging the caveats associated with hypotheses on the feeding ecology of extinct species, we take the combination of a small size, very long legs, and a comparatively short beak as indicative of an avivorous diet of *Aviraptor longicrus*. A short beak may facilitate a more powerful plucking of prey feathers, but the long tarsometatarsi of small avivorous hawks defy a straightforward explanation. Long legs may reduce the risk of injury for a bird catching comparatively large prey items in flight. However, not all avivorous birds of prey have long tarsometatarsi, and the adaptive significance of this feature in small hawks still needs to be assessed in functional morphological analyses.

The ecological attributes of avian prey preferred by diurnal raptors are poorly known. However, in forested environments, where most *Accipiter* species occur, foraging height and exposure appear to play major roles, with birds foraging near the ground being more accessible for diurnal birds of prey (Götmark and Post 1996). The species of *Accipiter* predominantly prey upon small forest passerines (Thiollay 1994), but the smallest extant sparrowhawk, *Accipiter superciliosus*, which is of similar size to *A. longicrus*, also hunts hummingbirds (Stiles 1978). Passerines may be particularly susceptible to predation by accipitrines because they vocalize from exposed song posts (Møller et al. 2008), which makes them more easily detectable by diurnal raptors foraging in forested environments.

Recognition of a small sparrowhawk-like raptor in the Rupelian of Poland is notable, because early Oligocene avifaunas of Europe include the earliest fossil records of both passerines and modern-type hummingbirds (Bochenski et al. 2013; Maxwell et al. 2016). The Eocene/Oligocene boundary marks an important faunal turnover in Europa known as the “Grande Coupure,” which was due to the closure of the Turgai Strait, a seaway separating Europe and Asia in the early Paleogene (Mayr 2009, 2011). Passerines probably dispersed into Europe during that geographic event, whereas hummingbirds evolved in Europe during the Eocene (Mayr 2009). We consider it possible that the occurrence of small-sized avivorous raptors in the early Oligocene of Europe was facilitated by a radiation of suitable prey, and *Aviraptor longicrus* may therefore represent an early example of avian predator/prey coevolution.

## Electronic supplementary material

ESM 1 (PDF 77.2 kb)
