# Supplementary material for: A tiny, long-legged raptor from the early Oligocene of Poland may be the earliest bird-eating diurnal bird of prey
Source: Naturwissenschaften. 2020 Oct 8;107(6):48. doi: 10.1007/s00114-020-01703-z (PMC7544617; doi:10.1007/s00114-020-01703-z)
Supplement: Supplementary file 1 — (PDF 77.2 kb) [file 114_2020_1703_MOESM1_ESM.pdf]

Supplementary Table 1. Length measurements (in mm) of major wing and leg bones and limb bone ratios for *Aviraptor longicrus*, n. gen. et sp. and selected extant Accipitridae and Falconidae. Specialized avivores among the extant species are highlighted with an asterisk (dietary preferences after Thiollay 1994; subfamily-level classification after Lerner and Mindell 2005 and Mindell et al. 2018).

species	sex	humerus	ulna	tibiotarsus	tarsometatarsus	hum:tmt	uln:tbt
			**New Oligocene accipitrid**				
Aviraptor longicrus	? [n=1]	37.5	47.2	45.3	43.8	0.86	1.04
			**Accipitridae: Accipitrinae**				
*Accipiter striatus**	♂ [n=1]	43.5	52.0	53.1	48.2	0.90	0.98
	♀ [n=1]	52.4	61.3	63.6	55.1	0.95	0.96
*A. nisus**	♂ [n=3]	50.0 ± 0.2	58.6 ± 0.1	58.9 ± 0.2	52.7 ± 0.4	0.95	0.99
	♀ [n=3]	59.9 ± 1.6	69.7 ± 2.0	70.0 ± 1.7	59.0 ± 1.9	1.02	1.00
*A. tachiro**	♂ [n=1]	57.3	64.6	71.5	59.3	0.97	0.90
	♀ [n=1]	70.7	82.3	90.2	71.7	0.99	0.91
*A. gularis**	♀ [n=2]	50.7 ± 0.4	60.5 ± 0.1	61.7 ± 0.4	52.2 ± 0.7	0.97	0.98
*A. cooperi**	♂ [n=2]	67.5 ± 0.9	73.1 ± 0.5	81.2 ± 1.9	64.5 ± 1.4	1.04	0.90
*A. rufiventris**	♀ [n=1]	59.6	69.2	70.1	56.5	1.05	0.99
	♀ [n=3]	78.1 ± 0.4	84.6 ± 1.4	93.6 ± 1.8	71.2 ± 0.9	1.10	0.90
A. gentilis	♂ [n=3]	88.6 ± 1.1	99.6 ± 1.6	102.3 ± 1.3	75.3 ± 0.9	1.18	0.97
	♀ [n=3]	103.0 ± 1.6	114.0 ± 1.3	114.7 ± 0.5	85.9 ± 1.5	1.20	0.99
Circus approximans	♂? [n=2]	105.3 ± 0.3	124.3 ± 0.1	109.1 ± 1.0	90.4 ± 1.1	1.16	1.14
	♀? [n=1]	115.6	137.1	119.0	94.1	1.23	1.15
C. aeruginosus	♂ [n=1]	100.5	120.2	103.8	81.9	1.23	1.16
	♀ [n=1]	108.9	129.1	115.2	92.1	1.18	1.12
			**Accipitridae: Buteoninae**				
Buteo buteo	♂ [n=3]	104.4 ± 2.1	122.1 ± 1.2	102.4 ± 0.	77.1 ± 2.3	1.35	1.19
	♀ [n=3]	109.2 ± 2.0	127.7 ± 2.6	107.3 ± 2.3	80.0 ± 1.2	1.37	1.19
B. magnirostris	♀ [n=1]	66.7	73.6	81.8	61.6	1.08	0.90
B. lineatus	? [n=1]	90.8	103.0	97.3	79.7	1.14	1.06
Buteogallus meridionalis	♂ [n=1]	118.3	137.4	129.0	110.3	1.07	1.07
Butastur teesa	? [n=1]	71.7	83.0	75.9	54.9	1.31	1.09
Milvus migrans	♂ [n=2]	119.9 ± 0.2	139.9 ± 1.7	85.0 ± 0.7	57.1 ± 0.1	2.10	1.65
	♀ [n=1]	120.0	139.5	85.7	56.7	2.12	1.63
Haliaeetus albilcilla	♂? [n=1]	205.0	237.6	156.0	97.2	2.11	1.52
	♀? [n=2]	232.0 ± 8.0	262.3 ± 6.9	170.9 ± 3.3	103.1 ± 2.9	2.25	1.53
			**Accipitridae: “transitory Accipitrinae”**				
Melierax canorus	? [n=1]	97.1	108.6	115.6	100.4	0.97	0.94
Harpagus bidentatus	? [n=1]	61.1	69.4	63.9	43.2	1.41	1.09
			**Accipitridae: Circaetinae**				
Circaetus gallicus	♂? [n=1]	160.0	185.7	127.3	88.7	1.80	1.46
	♀? [n=2]	168.5 ± 1.5	199.9 ± 2.1	131.4 ± 3.2	96.7 ± 2.8	1.74	1.52
			**Accipitridae: Aquilinae**				
Hieraaetus pennatus	? [n=1]	99.0	121.4	92.0	56.0	1.77	1.28
Aquila chrysaetos	? [n=1]	191.0	225.2	175.4	109.2	1.75	1.28
A. fasciata	♂? [n=1]	138.9 ± 0.5	167.1 ± 1.4	144.8 ± 0.1	98.6 ± 1.3	1.41	1.15
	♀? [n=1]	149.7	179.4	152.7	102.7	1.46	1.17
Nisaetus cirrhatus	♀ [n=1]	137.0	154.9	145.4	104.7	1.31	1.07
Spizaetus ornatus	♂ [n=1]	104.5	124.3	120.6	85.0	1.23	1.03
	♀ [n=1]	120.0	143.0	133.1	94.4	1.27	1.07
			**Accipitridae: Aegypiinae**				
Necrosyrtes monachus	♂ [n=1]	166.4	229.2	130.3	84.8	1.96	1.76
	♀ [n=1]	168.1	227.7	133.0	86.2	1.95	1.71
			**Accipitridae: Gypaetinae**				
Neophron percnopterus	? [n=1]	147.2	170.0	117.0	76.4	1.93	1.45
			**Accipitridae: Polyboroidinae**				
Polyboroides typus	♂ [n=1]	>105.5 (path.)	107.4	121.3	86.8	>1.22	0.89
			**Accipitridae: Perninae**				
Pernis apivorus	? [n=1]	104.4	116.0	86.0	51.0	2.04	1.35
Aviceda subcristata	? [n=1]	85.2	90.6	66.3	36.6	2.33	1.37
			**Accipitridae: Elaninae**				
Elanus caeruleus	? [n=1]	70.5	81.7	60.3	32.0	2.20	1.35
			**Falconidae**				
Falco sparverius	♂ [n=1]	42.2	47.2	50.3	35.9	1.18	0.94
F. tinnunculus	♂ [n=3]	52.4 ± 0.6	59.8 ± 0.8	57.8 ± 0.4	39.8 ± 0.4	1.32	1.03
	♀ [n=3]	55.3 ± 0.2	63.2 ± 0.4	60.2 ± 0.4	40.3 ± 0.6	1.37	1.05
